# Does environmental regulation improve residents' health? Evidence from China

**DOI:** 10.3389/fpubh.2023.973499

**Published:** 2023-01-27

**Authors:** Yunhe Pei, Hailin Chen, Zhenhai Liu, Haozhi Hu

**Affiliations:** ^1^School of Government, Sun Yat-sen University, Guangzhou, China; ^2^School of Economics and Trade, Guangdong University of Foreign Studies, Guangzhou, China; ^3^Department of Economics, Nanchang Hangkong University, Nanchang, China; ^4^Economics School, Zhongnan University of Economics and Law, Wuhan, China; ^5^Academy of Social Sciences, Zhongnan University of Economics and Law, Wuhan, China

**Keywords:** environmental regulation, residents health, CGSS, China, environment pollution

## Abstract

Environmental pollution is an important factor that harms public health, and environmental regulation is the policy instrument to govern pollution, so what impact does environmental regulation have on the public health? What are the mechanisms? To answer these questions, this paper constructs ologit model and uses China General Social Survey data for empirical analysis. The study found first that environmental regulation has a significant effect on improving the health level of residents, and this effect has been increasing with the passage of time. Second, the impact of environmental regulation on residents' health is different among residents with different characteristics. Specifically, the positive impact of environmental regulation on residents' health is stronger among residents with at least a university degree, residents with urban-registered residences, and residents living in economically developed areas. Third, the mechanism analysis found that environmental regulation can improve residents' health by reducing pollutant emissions and improving environmental quality. Finally, by introducing a cost benefit model, it was found that environmental regulation has a significant effect on improving the welfare level of individual residents and society as a whole. Hence, Environmental regulation is an effective means to improve residents' health, but when implementing environmental regulation, we should also pay attention to its negative impact on residents' employment and income.

## 1. Introduction

At present, environmental pollution is considered to be an important factor affecting human health, and it has become a common goal of countries across the world to adopt effective environmental regulation policies to reduce environmental pollution. Since Deng Xiaoping implemented the reform and opening-up policy in 1978, China has achieved rapid and sustainable economic development and has made remarkable achievements in the field of economic growth. At the same time, China has also become the country with the most serious environmental pollution problem in the world, and environmental pollution has also become a significant issue threatening the health of Chinese citizens. In recent years, this issue has also attracted the attention of many scholars around the world (1–3). Since the Twenty first century, China has gradually increased its attention to environmental issues. Especially since 2012, environmental governance has become an important indicator for assessing local government officials, and the environmental regulation policies implemented by central and local governments are also increasing. In 2007, China implemented the National Environment and Health Action Plan (2007–2015). Then, in 2013, the central government carried out the Action Plan for the Prevention and Control of Air Pollution. In April 2015, China implemented the Action Plan for the Prevention and Control of Water Pollution; subsequently, in May 2016, China implemented the Action Plan for the Prevention and Control of Soil Pollution. This trend of increasing environmental regulation shows the determination of the Chinese government to control environmental pollution.

Environmental regulation is an important means for the government to control environmental pollution. A large number of studies have shown that strengthening environmental regulation can effectively reduce environmental pollution (4, 5). Can environmental regulation improve residents' health effectively? If so, what are the mechanisms? At present, there are still few studies that determined this issue directly, and relevant studies are mainly analyzing it from the perspective of reducing environmental pollution. Yang and Chou (6) found that the New Jersey government significantly improved the local fetal health level by closing a coal-fired power plant following the implementation of the U.S. Clean Air Act. But this is only a case study, which is not representative enough and is not an empirical analysis. Applying time-series data of China, Zhou et al. (7) found that the more environmental governance policies and laws issued by the government, the fewer pollutant emissions in the country, which can reduce the nationwide expenditure on medical and healthcare. However, this study did not measure residents' health directly, and the sample size was too small. Taking the total number of patients in regional hospitals, Zhou et al. (8) used China's provincial panel data and found that strengthening environmental control can significantly reduce the number of patients in regional hospitals. In addition, Song et al. (9) found that environmental regulation can significantly improve the health level of regional human capital, thereby promoting regional economic development, but they did not analyze the mechanism by which environmental regulation affects the health level of human capital. Besides, a few studies have drawn other conclusions from different perspectives. Yan et al. (10) found that appropriate environmental regulation can promote employment and increase residents' income, which is conducive to improving citizens' health. However, when the intensity of environmental regulation is too strong, it will have a negative impact on employment and income and thus reduce residents' health. Peng et al. (11) pointed out that green innovation is the main mechanism for environmental regulation to reduce pollution and improve public health. Based on the “Porter hypothesis”, there is an inverted U-shaped relationship between environmental regulation and green innovation, which means that environmental regulation also has an inverted U-shaped effect on public health. However, the above studies only analyze from a single perspective and do not provide direct empirical evidence.

What are the effects and mechanisms of environmental regulation on residents' health? Obviously, this issue remains worthy of further study. As the largest developing country in the world, China is not only facing serious environmental pollution but is also one of the developing countries with the most experience in implementing environmental regulation policies. China's practical experience in environmental regulation also has important implications for other developing countries in the world seeking to control environmental pollution and improve the health of residents. This article uses the entropy method to construct a measurement indicator that reflects the strength of environmental regulation in China's provinces, then matches Chinese General Social Survey (CGSS) and China Statistical Yearbook data to empirically test the effects and mechanisms of environmental regulation on the health of Chinese residents. The main conclusions of this article are as follows: (1) from the overall effect, strengthening environmental regulation can significantly improve the residents' health; (2) environmental regulation mainly improves residents' health level by promoting innovation and reducing pollution, but it will reduce residents' health to a certain extent through reducing residents' income; and (3) the improvement effect of environmental regulation on residents' health is very significant among groups with different educational backgrounds and regions, but the effect is more intense among people with higher educational qualifications, living in urban registered residence and regions with higher economic development levels.

Compared to the existing studies, the possible contributions of this study are as follows. First, this study uses the entropy method to construct a variable that reflects the strength of regional environmental regulation and examines the direct effect of environmental regulation on residents' health, which can further deepen the understanding of health inequality and expand the research on welfare economics. Second, because we can only obtain microdata of individual health and macrodata of regional environmental regulation intensity during the empirical analysis, this study matched the micro-level survey data of residents' health found in the CGSS with information from multiple databases, such as the environmental regulation and the China Statistical Yearbook, when performing the empirical simulation. In addition to studying the overall effect of environmental regulation on residents' health, this study also analyzed the differences in the effect of environmental regulation on residents' health in different groups, which were divided by education level, registered residence location, and regional economic development level, and it extended the research scope from “absolute effect” to “relative effect,” which is conducive to understanding the inequality of China's environmental welfare. Third, this study analyzes the mechanisms of environmental regulation affecting residents' health from multiple channels, such as environmental pollution, innovation, and income, which is conducive to revealing the impact of environmental regulation on residents' health comprehensively.

The structure of this study is arranged as follows: the second part contains a literature review and theoretical hypothesis; the third part describes the research design, including the econometric model, variables, and data; the fourth part offers the empirical results and robustness test findings; the fifth part contains the analysis of heterogeneity and a discussion of the influence mechanism; the sixth part includes the cost–benefit analysis; and the seventh part presents the conclusions and offers policy recommendations of this study.

## 2. Literature analysis and theoretical hypothesis

### 2.1. Environmental regulation, environmental pollution, and residents' health

Since Gerking and Stanley (12) and Lipfert (13) incorporated environmental factors into the health production function. A large number of studies have confirmed that environmental pollution is an important factor affecting residents' health. Existing research shows that increasing environmental pollution will increase infant mortality (14), increase the probability of obesity and other diseases in children (15), increase the probability of residents suffering from cardiovascular disease and lung cancer (16), and improve the degree of mental depression in the elderly (17). There are even studies that have found that in areas with higher PM2.5 concentrations, the mortality of COVID-19-infected people is also higher (18).

Pollution is the main cause of environmental problems that harm residents' health. Therefore, reducing environmental pollution is the most important thing to eliminate the negative impact of environmental problems on residents' health. Environmental regulation is an important public policy for governments to control environmental pollution, and the impact of environmental regulation on environmental pollution is also a research hotspot. Existing studies show that there are three main ways for environmental regulation to reduce environmental pollution. First, environmental regulations increase the regulatory costs of enterprises, and high-pollution and high-emission enterprises have to bear higher environmental regulatory costs, which will lead to the bankruptcy and withdrawal of some high-pollution enterprises (5). Obviously, this will be conducive to the reduction of environmental pollution. Second, environmental regulations will force enterprises to increase R&D investment and carry out green innovation, thus reducing energy consumption and environmental pollution (19). Third, environmental regulations will encourage enterprises to carry out industrial transformation and upgrading, and high-pollution and high-emission industries will be gradually eliminated, which is also conducive to reducing environmental pollution (7). Many empirical research results also confirm this view. Wang et al. (20) built a DID model using the new Air Quality Standards implemented in China as a quasi-natural experiment to strengthen environmental regulation and found that strengthening environmental regulation could significantly improve the air quality by reducing the emission of sulfur dioxide and PM 2.5. Song et al. (21) made use of China's provincial panel data and found that environmental regulation can directly reduce environmental pollution by reducing enterprise pollution emissions through mandatory measures and indirectly reduce regional environmental pollution by promoting technological innovation and industrial upgrading. Moreover, the direct effect of environmental regulation on reducing environmental pollution is greater than the indirect effect. In addition, in areas with a higher economic development level, enterprises have stronger innovation abilities, and environmental regulations can better stimulate enterprises' innovation abilities and reduce environmental pollution, thus improving residents' health level.

It can be seen that environmental pollution has a significant negative impact on residents' health, and environmental regulation can effectively reduce environmental pollution. Therefore, theoretically, strengthening environmental regulation can improve residents' health by reducing environmental pollution (Hypothesis 1).

### 2.2. Environmental regulation, innovation, and public health

Innovation is an important technological driving force to improve residents' health. On the one hand, technological innovation can create a better living environment for residents, such as by reducing environmental pollution and improving life convenience, which is conducive to improving public health. On the other hand, part of the technological innovation can also be directly applied to the medical and health fields to promote the development of medical technology and improve the efficiency of the medical management system so as to improve the health level of residents (22). Camargo et al. (23) found that information technology can play an effective role in the prevention and control of Aedes aegypti and related diseases, thus improving the health level of residents. Georgescu et al. (24) also found that digital applications have effectively improved the efficiency of information delivery in healthcare systems and have played an important role in the fight against COVID-19 in European countries, thereby protecting the health of their populations.

The impact of environmental regulation on enterprise innovation has received extensive attention. The most popular Porter hypothesis holds that appropriate environmental regulations are conducive to driving enterprise innovation to alleviate the growing cost pressure brought by environmental regulations (25). Since then, many studies have explored the impact of environmental regulation on enterprise and regional innovation from the perspectives of the deterrent effect, resource compensation effect, and incentive and guidance effect (11, 26). The deterrent effect of environmental regulation means that environmental regulation will have a deterrent effect on enterprises' pollution behaviors by strengthening supervision and punishment. In this case, enterprises will be forced to actively develop new technologies, research new products, and develop new industries, thus improving the regional innovation level (27, 28). The resource compensation effect refers to the fact that environmental regulations can help enterprises get innovation returns faster and compensate innovation costs (25). Innovation is an investment with high risks and costs, which often requires enterprises to invest a large amount of research and development costs in the early stages. By implementing market incentive environmental regulation policies, the government can not only provide subsidies for enterprises' innovation and emission reduction behaviors directly but also establish market mechanisms, such as a carbon emission trading system, to help enterprises obtain innovation benefits as soon as possible (29). The above behaviors can effectively compensate for the R&D costs of enterprises, thus encouraging enterprises to innovate. The incentive and guidance effect refers to the fact that when the government implements environmental regulations, investors and consumers will pay more attention to the innovation ability of enterprises, especially the green innovation ability. In this case, enterprises are forced to pay more attention to improving their own innovation ability, and their innovation behaviors can also obtain more financial support from investors, which will encourage and guide enterprises to improve their innovation level (30).

It can be seen that innovation is an important factor affecting the health of residents, and promoting innovation is conducive to improving public health, while environmental regulation can improve the level of regional innovation through mechanisms such as the deterrence effect, the resource compensation effect, and the incentive and guidance effect. Therefore, environmental regulation can improve the health of residents by improving the innovation level of enterprises and regions (hypothesis 2).

### 2.3. Environmental regulation, income, and public health

Income level is an important economic factor affecting residents' health. On the one hand, increasing income levels can effectively improve residents' living environment, provide more food and other daily needs, reduce residents' living pressure and mental pressure caused by low income, and thus provide a more healthy living environment for residents (31). On the other hand, more income also means that people have a greater ability to treat diseases and thus avoid being harmed by them (32). Income level is generally regarded as a control variable affecting residents' health, and the positive impact of income on health has been confirmed by many empirical studies (33, 34). For example, Buckner et al. (35) found that children from low-income families usually have a higher probability of being exposed to violent environments, and violent experiences and environments will significantly damage children's mental health. Cai et al. (36) found that both absolute and relative income of residents have a significant positive impact on residents' health.

The impact of environmental regulation on residents' employment and income is mainly reflected in three aspects. The first is the cost effect, that is, environmental regulation will increase the institutional compliance cost of enterprises. In order to comply with the government's environmental regulation policies, enterprises have to invest more funds in improving production technology and strengthening green innovation to achieve energy conservation and emission reduction, which will inevitably increase the production cost of enterprises, squeeze the wage cost space of enterprises, and thus reduce employment or wages (37). The second is the output effect. Environmental regulation can effectively control the negative externalities of environmental pollution in production. The marginal cost of production can be increased by punishing enterprises' polluting behaviors or imposing environmental taxes, especially for some heavily polluting industries, enterprises often need to bear more environmental regulation costs. If the marginal production cost is higher than the marginal revenue due to environmental regulation, enterprises will reduce the production scale or even stop production. Enterprises will reduce the scale of production or even stop production, which will inevitably have a negative impact on residents' employment and income (38). The third is the employment structure effect. When faced with stronger environmental regulation, enterprises often need to invest more funds in the research of new technologies and new products, which will increase the demand for high-skilled labor but decrease the demand for low-skilled labor. However, in the labor market, the number of low-skilled labor is much larger than high-skilled labor, so there are far more low-skilled workers in the labor market than there are high-skilled workers, so more low-skilled workers will lose their jobs or have to have their wages cut (39).

It can be seen that income is an important economic factor affecting residents' health, and increasing income is conducive to improving residents' health, while environmental regulation may reduce labors' income through cost effect, output effect, and structural effect. Therefore, we can infer that strengthening environmental regulation will have a negative impact on residents' health by reducing their income level (Hypothesis 3).

## 3. Research design

### 3.1. Econometric model

As shown in Equation 1, to test the impact of environmental regulation on residents' health, this study built the following econometric model:


(1) $$\begin{array}{l} Health_{i,t}=\beta_{0}+\beta_{1}ER_{p,t}+\beta_{2}control_{i,t}+\eta_{c}+\varepsilon_{i,t} \end{array}$$

In Equation 1, *Health*_*i, t*_ reflects the health level of resident *i* in the time of *t*, *ER*_*p, t*_ is the intensity of environmental regulation of province *p*, where resident *i* is located in the time of *t*, *control*_*i, t*_ reflects a series of control variables, η_*c*_ is a control for the year fixed effects, and ε_*i, t*_ is a random disturbance term.

This study needed to focus on the regression coefficient of *ER*, namely, β_1_. When β_1_ is significantly positive, it indicates that environmental regulation can improve residents' health level. Conversely, when β_1_ is significantly negative, it shows that environmental regulation will reduce residents' health level.

### 3.2. Variable selection

#### 3.2.1. Dependent variable

The dependent variable used in the study was the self-reported health level of residents (*Health*), which is a comprehensive judgment of interviewees' health situation according to many factors, including disease severity, family disease history, and health stability. This measurement method can meet requirements for the sufficiency of psychometrics and the reliability and validity of statistics. Therefore, previous studies on residents' health adopted similar methods (7). This study selects the item of “what do you think of your physical health?” in the CGSS questionnaire to measure the self-reported health of residents (*Health*). According to the respondents' answers, we reset codes 4, 3, 2, 1, and 0 to correspond to “very good,” “good,” “fair,” “bad,” and “very bad,” respectively; the larger the value, the better the self-reported health (*Health*).

#### 3.2.2. Independent variable

Environmental regulation (*ER*) is the core independent variable. To reflect the intensity of formal environmental regulation more accurately, this study referred to the method used by Fu and Li (40), which was adjusted to fit the purpose of this study. Specifically, based on the scale of various pollutant emissions in China and the availability of data, this study selected four single indicators, namely, the wastewater discharge compliance rate, SO_2_ removal rate, smoke (dust) removal rate, and solid waste comprehensive utilization rate, to build a comprehensive measurement system of environmental regulation. First, we carried out linear standardization of the four single indicators according to the method shown in Equation 2, that is, the value of each indicator was standardized as (0, 1) so as to eliminate differences in the measurement of different indicators.


(2) $${\text{PR}}_{\text{ijt}}{}^{\prime }\text{=}[{\text{PR}}_{\text{ijt}}-\text{min}({\text{PR}}_{\text{j}})]\text{/}[\text{max}({\text{PR}}_{\text{j}})-\text{min}({\text{PR}}_{\text{j}})]$$

In Equation 2, *i* represents the province (I = 1,2,3,..., 30), *j* represents the single indicator of environmental regulation (*j* = 1,2,3,4), *t* represents the year, *PR*_*ijt*_is the original value of each single indicator, *max* (*PR*_*j*_) and min (*PR*_*j*_) are the annual maximum and minimum values of the four single indicators in each province, and $PR_{ijt}^{{}^{\prime }}$ is the standardized value of each single indicator.

Second, we calculated the weight of each single index (ω_*ij*_). For different provinces, the proportion of various pollutants is quite different, so the intensity and weight given by environmental regulations to different pollutants also vary. Adjusting the weight of each single index value can reflect the difference in the governance of major pollutants in each province. The weight of each single index may be calculated as follows:


(3) $$\omega_{ijt}=(\frac{E_{ijt}}{\sum E_{ijt}})/(\frac{Y_{i}t}{\sum Y_{it}})$$

In Equation 3, ω_*ijt*_ is the weight of pollutant *j* in province *i*, *E*_*ijt*_ is the emission of pollutant *j* in province *i*, _*ijt*_ is the total emissions of similar pollutants in China, *Y*_*it*_ is the industrial added value of province *i*, and _*it*_ is the national industrial added value. After obtaining the annual weight of each single indicator, we calculated the average value of the weight in the selected year.

Finally, using the standardized value and average weight of each single index, we could measure the intensity of environmental regulation in each province using the following equation:


(4) $$Env_{i}=\frac{1}{4}\sum_{j=1}^{4}\bar{\omega_{ij}}*PR_{ijt}{}^{\prime }$$

The larger the value of *Env*_*i*_, the stronger the intensity of environmental regulation.

#### 3.2.3. Control variables

Referring to existing studies, residents' own factors and social factors were considered in the control variables. In terms of the residents' own factors, we considered the residents' age, gender, and marital status as the control variables. Existing research shows that there is a non-linear relationship between age and health: before the age of 30 years, a positive relationship exists, while after the age of 30 years, health declines with age with an accelerating trend (41). Furthermore, women's self-evaluation of health is often lower than that of men (42). Moreover, marriage can promote a good lifestyle and improve the health status of residents (43, 44).

Social factors usually include the economic development level and population of the region. The existing literature shows that, from the perspective of provincial- or prefecture-level units, the self-evaluation of health status of residents in socio-economically developed areas is significantly better than that of residents in underdeveloped areas (45). The increase in population will lead to congestion effects, such as rising commuting costs and environmental degradation, and can reduce the health level of residents (46). In addition, with the development of green finance in recent years, some scholars have pointed out that green finance can promote industrial integration; reduce the proportion of the secondary industry; expand the scale of the tertiary industry; and improve the competitiveness of green industries to achieve energy conservation, emission reduction, and environmental improvement (47). The definitions of related variables are presented in Table 1.

**Table 1:** Definitions of variables.

**Variable**	**Symbol**	**Measure of variables**
Self-reported health	Health	Self-reported of physical health in CGSS questionnaire; the larger the value, the better the self-evaluated health
Environmental Regulation	ER	The intensity of environmental regulation calculated by the entropy method
Age	AGE	Actual age of residents' questionnaire
Gender	GEN	Female = 0, male = 1
Educational status	EDU	Below primary school = 0, primary school = 1, junior high school = 2, senior high school = 3, college and above = 4
Marital status	MAR	Unmarried, divorced, or widowed = 0; married, cohabiting, or having a permanent partner = 1
Green finance index	GFIN	Provincial digital financial index
GDP per capita	GDPPC	The real GDP per capita at the provincial level takes the natural logarithm
Population size	LnPOP	The provincial population of the domicile takes the natural logarithm
Change of industrial structure	CHIS	The change value of industrial structure measured by the vector angle method (47)

### 3.3. Sample selection and data source

Among the data used in this study, the microlevel data come from the CGSS, and the original macrolevel economic data come from the China Statistical Yearbook and provincial statistical yearbooks. Among them, CGSS survey data from 30 provinces in total across 8 years (2005, 2008, 2010, 2011, 2012, 2013, 2015, and 2017) were used, and a total of 80,046 samples were finally selected to build the dataset. The actual per-capita gross domestic product (GDP) and population values were taken as natural logarithms in the empirical analysis. Because health indicators have only been included in CGSS survey data since 2005, the price of the actual per-capita GDP was based on 2005 information.

Table 2 shows the descriptive statistics of relevant variables in this study. It can be seen from Table 2 that the average health level of residents in this study was 2.578, the minimum value was 0, and the maximum value was 4, which are basically consistent with the results of previous research and investigations. In addition, the average value of *Env* was 0.366, the minimum value was 0.079, and the maximum value was 1.49, indicating that there are differences in environmental regulations in the sample areas. Also, the mean age was 48.126 years, the minimum age was 17 years, and the maximum age was 103 years, indicating that the majority of the sample population was middle-aged or older people. At the regional level, the regional per-capita GDP, green financial development, and regional population were heterogeneous in different provinces.

**Table 2:** Description statistics of variables.

**Variable**	***N***	**Mean**	**SD**	**Min**	**Max**
AGE	80392	48.058	16.206	17	103
EDU	80390	2.032	1.248	0	4
MAR	80392	0.797	0.402	0	1
ER	80392	0.366	0.17	0.079	1.49
GEN	80392	0.485	0.5	0	1
Health	80392	2.581	1.132	0	4
CHIS	80392	6.619	0.347	5.949	7.607
GFIN	80392	0.172	0.114	0.053	0.759
GDPPC	80392	10.24	0.583	8.557	11.558
LnPOP	80392	8.423	0.597	6.335	9.404

## 4. Empirical results

### 4.1. Benchmark regression

As the dependent variable in this study was an ordered discrete variable, if ordinary least squares regression were to be adopted, it may cause estimation bias in the regression results. Therefore, this study used the ologit model for benchmark regression, and the relevant regression results are shown in Table 3. M3-1 only took environmental regulation as the explanatory variable in the model, M3-2 added other control variables to the model, M3-3 controlled the fixed effect of the year, and M3-4 further clustered data at the individual level. The results show that, in the four models in Table 3, the strength of environmental regulation had a positive impact on the resident's health at the significance level of 1%, that is, strengthening environmental regulation can significantly improve the probability of residents' becoming healthier. In addition, the impact of control variables on residents' health was consistent with findings of theoretical analysis and significant at the level of 1%, which also shows the effectiveness of model setting and variable selection.

**Table 3:** Regression results.

**Variable**	**M3-1**	**M3-2**	**M3-3**	**M3-4**
	**Health**	**Health**	**Health**	**Health**
**ER**	**0.255** ^***^	**0.550** ^***^	**0.784** ^***^	**0.768** ^***^
	**(0.037)**	**(0.042)**	**(0.045)**	**(0.065)**
AGE		−0.040^**^	−0.041^***^	−0.039^***^
		(0.000)	(0.000)	(0.002)
GEN		0.270^***^	0.277^***^	0.177^***^
		(0.013)	(0.013)	(0.014)
EDU		0.196^***^	0.190^***^	0.185^***^
		(0.006)	(0.006)	(0.012)
MAR		0.073^***^	0.055^***^	0.058^**^
		(0.016)	(0.016)	(0.022)
GFIN		−0.380^***^	0.566^***^	0.559^***^
		(0.112)	(0.118)	(0.114)
LnGDPPC		0.65^***^	0.672^***^	0.672^***^
		(0.022)	(0.029)	(0.037)
LnPOP		0.286^***^	0.201^***^	0.201^***^
		(0.013)	(0.013)	(0.019)
CHIS		0.302^***^	−0.612^***^	−0.612^***^
		(0.051)	(0.058)	(0.069)
*Year*	N	N	Y	Y
*Pro*	N	N	N	Y
*N*	80392	80390	80390	80390
*Pseudo*−*R*^2^	0.0002	0.0635	0.0813	0.0852

The parentheses are standard errors, and the standard errors of the model are clustered to the individual level. ^***^, ^**^, and ^*^ indicate significance at the 1%, 5%, and 10% levels, respectively. Year and Pro means the fixed effect of year and province, respectively.

### 4.2. Robustness test

The above empirical results show that regional environmental regulation has a significant positive impact on residents' health. Considering that randomness may exist in the empirical results, this section tested their robustness by using the following three methods.

First, we took the first-order lag terms of the variable of environmental regulation as explanatory variables for regression, which can not only alleviate the endogenous problem to a certain extent but also test the dynamic effect of environmental regulation on residents' health. As shown in M4-1, when we use the first-order lag term of environmental regulation as the independent variable, environmental regulation still has a positive effect on resident's health at the significance level of 1%, which also indicates the robustness of the empirical results. In addition, we also find that the impact coefficient of environmental regulations in M4-1 is smaller than that in M3-4, indicating that the impact of environmental regulations on residents' health will be weakened after 1 year.

Second, we tested its robustness by changing the regression model. In this part, we used the probit model to regress, and the result is shown in M4-2. Specifically, the result shows that the coefficient of environmental regulation is significantly positive at the level of 1%, which also indicates that strengthening environmental regulation is conducive to improving the probability of residents obtaining a higher health level.

Third, we changed the robust clustering standard from the individual level to the provincial level, and the results are shown in M4-3. It can be seen from the result that the coefficient of environmental regulation remained significantly positive at the level of 1%, which also confirms that the benchmark regression results mentioned above are robust.

Finally, this part also collected the number of environmental trial cases in each province from the environmental judicial judgements in China and standardized the number of cases according to the local population (unit: 100,000) as an alternative measurement of the intensity of environmental regulation to test the robustness of the regression. As shown in M4-4 in Table 4, the regression coefficient is positive and significant at the level of 1%, which indicates that strengthening environmental justice also has a significant positive effect on residents' health levels.

**Table 4:** Results of robustness test.

**Variable**	**M4-1**	**M4-2**	**M4-3**	**M4-4**
	**Health**	**Health**	**Health**	**Health**
**ER**		**0.456** ^***^	**0.764** ^***^	**0.177** ^***^
		**(0.034)**	**(0.134)**	**(0.018)**
**ER1**	**0.518** ^***^			
	**(0.042)**			
*Control*	Y	Y	Y	Y
*Year*	Y	Y	Y	Y
*Pro*	Y	Y	Y	Y
*N*	80390	80390	80390	28111
*Pseudo*−*R*^2^	0.0819	0.0822	0.0837	0.0827

The parentheses are standard errors, and the standard errors of the model are clustered to the individual level. ^***^, ^**^, and ^*^ indicate significance at the 1%, 5%, and 10% levels, respectively. Control means the control variables, and Year and Pro means the fixed effect of year and province, respectively.

### 4.3. Heterogeneity analysis

Considering China's vast territory, the degree of economic development in different regions is quite different, and the differences among residents will also lead to a varying impact on their health. Different groups of residents have different abilities to use environmental resources and avoid the negative externalities of the environment. It is necessary to further study whether there are significant differences in the impact of environmental regulation on the health of different groups of residents. In this part, the samples were grouped according to education level (having a university degree or not), difference in registered residence location (rural registered residence vs. urban registered residence), economic development level of the residence region (taking the average value of regional per-capita GDP as the standard), and the intensity of environmental regulation (taking the average value of) and the heterogeneity of the impact of environmental regulation on residents' health was tested, respectively.

The results of the grouping test are shown in Table 5. Although in different groups, strengthening environmental regulation can significantly improve the probability of residents obtaining a higher level of health, this effect still varies among the groups. M5-1 and M5-2 are the regression results of the groups with less than a college education and with a college education or above, respectively. The results show that, in the group with more education, the effect of environmental regulation on the improvement of residents' health was stronger. This is because the group with higher education has a better ability to use environmental resources and avoid the negative externalities of the environment. Therefore, when environmental regulation improves the living environment, highly educated people have greater motivation and ability to use the environment to improve their health, such as by carrying out fitness activities.

**Table 5:** Heterogeneity tests.

**Variable**	**M5-1**	**M5-2**	**M5-3**	**M5-4**	**M5-5**	**M5-6**
	**Health**	**Health**	**Health**	**Health**	**Health**	**Health**
**ER**	**0.654** ^***^	**0.527** ^**^	**0.629** ^***^	**0.643** ^***^	**0.519** ^***^	**1.024** ^***^
	**(0.038)**	**(0.281)**	**(0.071)**	**(0.154)**	**(0.068)**	**(0.106)**
*Control*	Y	Y	Y	Y	Y	Y
*Year*	Y	Y	Y	Y	Y	Y
*Pro*	Y	Y	Y	Y	Y	Y
*N*	67549	12841	41798	38588	43231	37159
*Pseudo*−*R*^2^	0.0721	0.0754	0.0823	0.0857	0.0829	0.0846

The parentheses are standard errors, and the standard errors of the model are clustered to the individual level. ^***^, ^**^, and ^*^ indicate significance at the 1%, 5%, and 10% levels, respectively. Control means the control variables, and Year and Pro means the fixed effect of year and province, respectively.

M5-3 and M5-4 are the regression results of the groups with urban-registered residences and rural-registered residences, respectively. The results show that the positive effect of environmental regulation on residents' health among rural residents is stronger than it is among urban residents. This is because China's environmental pollution is mainly concentrated in urban areas, and environmental regulation has a greater impact on reducing urban environmental pollution.

M5-5 and M5-6 are the regression results of samples in regions with lower and higher economic development levels, respectively. The results show that, in regions with a higher level of economic development, the positive effect of environmental regulation on residents' health is stronger. This is because regions with more economic development have stronger technical levels and innovation abilities. Thus, when faced with environmental regulation, their ability to reduce environmental pollution through green innovation and economic transformation is also greater.

### 4.4. Mechanism test

Theoretical analysis shows that strengthening environmental regulation may improve the health of residents by reducing environmental pollution, promoting innovation and raising the level of residents' income. To verify this, this study constructed the following econometric models:


(5) $$\begin{array}{c} M=\lambda_{0}+\lambda_{1}Env_{p}+\lambda_{2}X_{p}+\eta_{c}+\delta_{p} \end{array}$$

(6) $$\begin{array}{c} Health_{i}=\gamma_{0}+\gamma_{2}M+\gamma_{3}control_{i}+\eta_{c}+\tau_{i} \end{array}$$

Equation 5 is an econometric model to test the impact of environmental regulation on variables, where *p* is the province; *M* reflects the mechanisms, namely, pollution, innovation, and income; *Env*_*p*_ is the variable measuring the intensity of environment regulation; and *X*_*p*_ is a group of control variables. In Equation 6, the variable of environmental pollution was added to the model shown in Equation 1 as an explanatory variable to test the impact of mechanism on residents' health. If in Equation (5), environmental regulation has a significant impact on the mechanism variable (M), and in Equation (6), the mechanism variable (*M*) also has a significant impact on residents' health, it can be shown that M is one of the mechanisms by which environmental regulation affects residents' health.

Table 6 shows the regression results of the mechanism test of environmental pollution. Taking the per-capita SO_2_ emission, per-capita nitrogen oxide emission, and per-capita smoke and dust emission (dust) of each province as the dependent variables to measure the degree of environmental pollution and taking the natural logarithm of the dependent variables, we used the model shown in Equation 5 for regression; the regression results are shown in M6-1 to M6-3, respectively, and reveal that environmental regulation has a significant effect on reducing the emissions of SO_2_, nitrogen oxides, and dust at the 1% significance level, which also verifies hypothesis. In M6-4 to M6-6, SO_2_, nitrogen oxides, and dust were added as explanatory variables to the model shown in Equation 6 for regression, and the results show that, in the three models, environmental pollution significantly reduced the health levels of residents at the significance level of 1%. These results show that strengthening environmental regulation can improve residents' health significantly by reducing environmental pollution, which also verifies hypothesis 1.

**Table 6:** Mechanism test (1).

**Variable**	**M6-1**	**M6-2**	**M6-3**	**M6-4**	**M6-5**	**M6-6**
	**SO**_2_	**NO**	**Dust**	**Health**	**Health**	**Health**
ER	−1.269^***^	−0.648^***^	−0.649^***^			
	(0.023)	(0.021)	(0.018)			
SO_2_				−0.021^***^		
				(0.001)		
NO					−0.019^***^	
					(0.002)	
Dust						−0.038^***^
						(0.004)
Control	Y	Y	Y	Y	Y	Y
*Year*	Y	Y	Y	Y	Y	Y
*Pro*	Y	Y	Y	Y	Y	Y
*N*	64023	52381	63623	64023	52381	63623
*R* ^2^	0.286	0.319	0.321	0.087	0.094	0.082

The parentheses are standard errors, and the standard errors of the model are clustered to the individual level. ^***^, ^**^, and ^*^ indicate significance at the 1%, 5%, and 10% levels, respectively. Control means the control variables, and Year and Pro means the fixed effect of year and province, respectively.

Table 7 shows the test results of the other two mechanisms. In M7-1, using the natural logarithm of per capita income as the dependent variable and the intensity of provincial environmental regulation as the core independent variable, the regression result shows that environmental regulation reduces the level of resident income at the significance level of 1%. In M7-2, the residents' income is added into the model shown in Equation 6 as an independent variable, and the regression result shows that the residents' income has a significant positive impact on the residents' health at the 1% significance level. Using the number of patents granted per 10,000 people in each province to measure regional innovation and taking its natural logarithm as the independent variable, M7-3 finds that environmental regulation can significantly promote regional innovation at the 1% significance level. Similarly, by adding innovation as an independent variable to Equation 6, the regression result in M7-4 shows that regional innovation has a significant positive impact on residents' health at the 1% significance level. The above results also indicate that environmental regulation can not only improve residents' health by promoting innovation but can also reduce residents' health by reducing income, which also proves hypothesis 2 and hypothesis 3.

**Table 7:** Mechanism test (2).

**Variable**	**M7-1**	**M7-2**	**M7-3**	**M7-4**
	**Income**	**Health**	**Innovation**	**Health**
ER	−0.633^***^		0.131^***^	
	(0.107)		(0.089)	
Income		0.026^***^		
		(0.002)		
Innovation				0.035^***^
				(0.008)
Control	Y	Y	Y	Y
*Year*	Y	Y	Y	Y
*Pro*	Y	Y	Y	Y
*N*	28111	28111	300	28111
*R* ^2^	0.081	0.087	0.294	0.082

The parentheses are standard errors, and the standard errors of the model are clustered to the individual level. ^***^, ^**^, and ^*^ indicate significance at the 1%, 5%, and 10% levels, respectively. Control means the control variables, and Year and Pro means the fixed effect of year and province, respectively.

### 4.5. Cost–benefit analysis

The above empirical results show that environmental regulation has a significant positive effect on residents' health; however, the implementation of environmental regulation will also bring costs and economic losses and even place financial pressure on some local governments. Hence, local governments also need to balance economic development and environmental protection when implementing environmental regulation policies. Therefore, it is also necessary to take the cost–benefit comparison into account when evaluating the effects of environmental regulation policies (48, 49). Existing studies have shown that environmental regulation can effectively improve environmental quality and then have a positive impact on improving public health, reducing infant mortality, improving labor productivity, and reducing the crime rate (50–53). Based on the cost–benefit analysis method, this part tests the economic and social costs and benefits brought by environmental regulation. Referring to the exiting literature, we use health-related expenditures to measure economic costs and benefits (2), and traffic accidents and deaths to measure social costs and benefits (54). To test the economic and social effects of environmental regulation, we constructed the following econometric models:


(7) $$\begin{array}{c} {\text{C}}_{\text{p},\text{t}}={\text{β}}_{0}+{\text{β}}_{1}\text{En}{\text{v}}_{\text{p}}+{\text{β}}_{2}\text{contro}{\text{l}}_{\text{i}}+{\text{η}}_{\text{c}}+{\text{ε}}_{\text{i}} \end{array}$$

In Equation (7), *C*_*p, t*_ is the variable to reflect social and economic costs and benefits. To test the economic costs and benefits, we take the sum of social security, medical, and health expenditures as the financial expenditures of local governments in China's provinces as the macro-level health care costs [marked as MAHC_(p,t)] and use the per-capita expenditures of social security, medical, and health as the microlevel healthcare costs [marked as MIHC_(i,t)]. The regression results are shown in Table 8. In M8-1, environmental regulation has a significant negative effect on the total fiscal expenditure in medical and healthcare in each province at the level of 1%, and similarly, in M8-2, environmental regulation also has a significant negative effect on the per capita medical and healthcare expenditure at the level of 1%. The above results indicate that environmental regulation can effectively reduce medical and healthcare expenditure from both macro- and microlevels, thus bringing economic benefits to local government and individuals.

**Table 8:** Test results of cost–benefit analysis.

**Variable**	**M8-1** ** MAHC**	**M8-2** ** MIHC**	**M8-3 Accidents**	**M8-4** ** Injuries**
ER	−0.189^***^	−0.3226^***^	−0.500^***^	−0.481^***^
	(0.013)	(0.107)	(0.040)	(0.044)
Control	Y	Y	Y	Y
*Year*	Y	Y	Y	Y
*Pro*	Y	Y	Y	Y
*N*	300	28456	300	300
*R^2^*	0.374	0.109	0.295	0.307

The parentheses are standard errors, and the standard errors of the model are clustered to the individual level. ^***^, ^**^, and ^*^ indicate significance at the 1%, 5%, and 10% levels, respectively. Control means the control variables, and Year and Pro means the fixed effect of year and province, respectively.

In addition, this part also uses the number of traffic accidents and the number of injured people caused by traffic accidents in each province of China to test the costs and benefits brought by environmental regulation. As shown in M8-3 and M8-4, environmental regulations have significantly reduced the number of traffic accidents and the number of injuries in traffic accidents at the level of 1%, and this also shows that environmental regulations can effectively increase social benefits. Of course, we can also analyze the economic and social costs and benefits of environmental regulation from other dimensions, such as economic development, tax revenue, corruption, and crime rate, but since these are not the core issues concerned in this study, this study only analyzes the above four indicators.

## 5. Conclusion and suggestions

This study uses CGSS data to match the China Statistical Yearbook and provincial statistical yearbooks to construct a database and selects eligible residents as research samples to investigate the impact of environmental regulation on residents' health levels. This study also has some meaningful findings. First, environmental regulation has a significant effect on improving residents' health on the whole. Second, the regression results of the mechanism analysis show that strengthening environmental regulation can significantly improve residents' health by reducing pollution and promoting innovation, but it will harm residents' health by reducing their income. Third, although the positive effect of environmental regulation on residents' health is significant among residents with different characteristics, it is stronger among residents with higher education experience or who are living in urban or areas with a higher level of economic development. Finally, through a cost–benefit analysis, it is found that environmental regulation can significantly reduce economic costs and increase social benefits.

The conclusions of this study also have some policy implications for the government to strengthen environmental governance, reduce environmental pollution, and improve residents' health. First of all, residents' health is an important part of social welfare, and environmental pollution has a significant negative impact on residents' health, which shows that governments should pay more attention to controlling environmental pollution. For a long time, many countries, including China, have considered economic growth to be the primary goal of development. In order to attract more foreign investments, the government of China continuously reduced environmental protection standards and neglected the impact of environmental pollution on residents' health. It formed a development model of high energy consumption and high pollution emissions, which has had a serious negative impact on the health of local residents and damaged the overall social welfare level of the country. Therefore, to promote regional sustainable development, China and other countries going forward should adjust their development models and pay more attention to environmental protection by adjusting their development goals, value orientation, and policies and by abandoning the traditional development model that considers economic growth as its single goal. Second, environmental regulation is an important means for the government to reduce environmental pollution. Moreover, it can also provide a better living environment for residents by reducing environmental pollution and promoting innovation so as to improve the health levels of residents. Other countries could consider making efforts in the areas of environmental legislation and strengthening environmental law enforcement. By implementing stricter environmental regulation policies and penalties, it may promote enterprises to implement green innovation and reduce environmental pollution. More importantly, governments should strictly punish those who violate environmental protection laws and regulations and should take incentive measures to promote enterprises to implement green innovation to reduce environmental pollution. In addition, environmental regulations will also damage residents' health by reducing their income. Therefore, when implementing environmental regulations, the government should pay more attention to low-skilled people and low-income groups and take effective measures to increase their income so that they can avoid being harmed by environmental regulations. Finally, the impact of environmental regulation on residents' health level is also heterogeneous. Residents' education level, living area, and local economic development level may affect the improvement effect of environmental regulation on residents' health. Therefore, countries should also pay more attention to developing public education and improving the education level of residents while implementing environmental regulation. The government also needs to adopt measures to narrow the development gap between urban and rural areas so that more people in underdeveloped areas can also enjoy the positive spillover effect of environmental regulation.

There are some limitations in this study, which will be further improved in future research. First, due to limitations on the availability of data, this study used provincial data to measure the intensity of environmental regulation and matched it with microlevel survey data, which failed to reflect the differences in the intensity of environmental regulation among regions within each province. In future, we will try to match the data of prefecture cities with microlevel data. Second, in the mechanism analysis, we considered the effects of environmental regulation on reducing environmental pollution, promoting innovation, and reducing residents' income. In future research, we will try to reveal the mechanisms of environmental regulation that affect residents' health more comprehensively and provide more effective policy suggestions for improving residents' health through the implementation of environmental regulation.

## Data availability statement

The original contributions presented in the study are included in the article/supplementary material, further inquiries can be directed to the corresponding author.

## Author contributions

YP is responsible for data collecting and empirical analysis and undertakes the main work of paper writing. HC is responsible for literature collecting and theoretical framework building. ZL contributes to the resources, data analysis, and original draft preparation. HH contributes to the study's organization, the research methods, and performed the review and editing of the manuscript. All authors contributed to the article and approved the submitted version.
